# Invasive Pulmonary Aspergillosis in Coronavirus Disease 2019 Patients Lights and Shadows in the Current Landscape

**DOI:** 10.3390/arm91030016

**Published:** 2023-05-08

**Authors:** Stavros Tsotsolis, Serafeim-Chrysovalantis Kotoulas, Athina Lavrentieva

**Affiliations:** 1 Medical School, Aristotle University of Thessaloniki, Leoforos Agiou Dimitriou, 54124 Thessaloniki, Greece; 2 1st ICU, General Hospital of Thessaloniki “Georgios Papanikolaou”, Leoforos Papanikolaou, 57010 Thessaloniki, Greece

**Keywords:** invasive pulmonary aspergillosis, COVID-19, SARS-CoV-2, critically ill, review, diagnostic algorithm, treatment options

## Abstract

**Highlights:**

What are the main findings?

- A definite diagnosis of invasive pulmonary aspergillosis is elusive in patients with severe COVID-19.
- Experimental and clinical data indicate that delayed initiation of antifungal therapy could be detrimental to IA.

What is the implication of the main finding?

- The persistence of a respiratory co-infection in SARS-CoV-2 patients despite the administration of broad-spectrum antibiotics should lead to the pursuit of the confirmation or exclusion of IPA, especially in those patients who present risk factors for invasive pulmonary aspergillosis.
- Early treatment should be initiated, even in the absence of a definite diagnosis, when clinical suspicion is high.

**Abstract:**

Invasive pulmonary aspergillosis (IPA) presents a known risk to critically ill patients with SARS-CoV-2; quantifying the global burden of IPA in SARS-CoV-2 is extremely challenging. The true incidence of COVID-19-associated pulmonary aspergillosis (CAPA) and the impact on mortality is difficult to define because of indiscriminate clinical signs, low culture sensitivity and specificity and variability in clinical practice between centers. While positive cultures of upper airway samples are considered indicative for the diagnosis of probable CAPA, conventional microscopic examination and qualitative culture of respiratory tract samples have quite low sensitivity and specificity. Thus, the diagnosis should be confirmed with serum and BAL GM test or positive BAL culture to mitigate the risk of overdiagnosis and over-treatment. Bronchoscopy has a limited role in these patients and should only be considered when diagnosis confirmation would significantly change clinical management. Varying diagnostic performance, availability, and time-to-results turnaround time are important limitations of currently approved biomarkers and molecular assays for the diagnosis of IA. The use of CT scans for diagnostic purposes is controversial due to practical concerns and the complex character of lesions presented in SARS-CoV-2 patients. The key objective of management is to improve survival by avoiding misdiagnosis and by initiating early, targeted antifungal treatment. The main factors that should be considered upon selection of treatment options include the severity of the infection, concomitant renal or hepatic injury, possible drug interactions, requirement for therapeutic drug monitoring, and cost of therapy. The optimal duration of antifungal therapy for CAPA is still under debate.

## 1. Introduction

Fungal diseases range from relatively minor superficial and mucosal infections to severe, life-threatening systemic infections. Delayed diagnosis and treatment could result in serious consequences for patient outcomes and could be associated with high medical costs [1,2].

The global burden of fungal diseases is increasing, given the expanding number of patients at risk for these infections, including people with human immunodeficiency virus (HIV), hematological and solid organ transplants recipients, patients with malignancies, patients receiving immunomodulation therapy and elderly patients [3]. The Global Action Fund for Fungal Infections (GAFFI) estimates that the prevalence of fungal infections ranges between 1.6% and 3.6% worldwide [4]. The Centers for Disease Control and Prevention (CDC) estimated that fungal diseases cost more than USD 7.2 billion in direct medical costs in 2017 based on administrative coding data [5]. The total costs are likely to be significantly higher when counting indirect and societal costs [6]. A multicenter French study describing trends in invasive fungal infections (IFIs) based on hospital discharge data found a 4.4% increase in invasive aspergillosis (IA) each year during a decade [7]; similar increases have been noted in other publications [6,8,9]. Interestingly, IA accounted for nearly 20% of all IFIs among organ transplant recipients, second only to invasive candidiasis [10,11]. IA caused by *Aspergillus* species (*A. fumigatus*, *A. niger*, *A. flavus*, *A. terreus*) carries a high overall mortality rate (30 to 95%), even if the disease is diagnosed early and despite the prompt use of antifungal treatment approaches [12]. Invasive pulmonary aspergillosis (IPA) is a frequent complication of critically ill patients with H1N1 virus infection and severe respiratory failure [13,14,15].

The ongoing pandemic of Coronavirus disease 2019 (COVID-19), caused by severe acute respiratory syndrome coronavirus 2 (SARS-CoV-2) may increase the burden of IA and cause several challenges regarding diagnostic and treatment approaches [16]. Damaged respiratory epithelium, dysfunctional mucociliary clearance, and local immune suppression—all features present in COVID-19—were demonstrated to be key pathophysiological factors contributing to the development of IPA [14].

In this brief review, we discuss the current state of the art, regarding the diagnosis and treatment of COVID-19-associated pulmonary aspergillosis (CAPA) in the ICU. All abbreviations used, are expanded in Appendix A, Table A1.

## 2. Incidence, Risk Factors and Outcome of IPA in Patients with SARS-CoV-2 Infection

### 2.1. Incidence

IPA presents a known risk to critically ill patients with SARS-CoV-2. Quantifying the global burden of IPA in SARS-CoV-2 is extremely challenging because of the presence of a number of confounding factors:

- IPA frequently manifests with nonspecific symptoms and is not routinely suspected;
- Respiratory deterioration is considered to be caused by bacterial co-infection rather than fungal infection;
- Diagnosis of IA, frequently, requires invasive tissue specimens collection;
- Histopathologic identification is challenging;
- Cross-reaction of fungal antibody tests may exist;
- Lack of routine surveillance for IA is common.

According to recently published data from European centers, the incidence of CAPA varies between 20–35% of all mechanically ventilated patients (referring to both possible or probable diagnosis) [16,17,18,19,20,21]. Van Arkel et al. observed a high incidence of IPA in a cohort of 31 critically ill patients with SARS-CoV-2; eleven ICU patients developed a secondary infection, of whom six (19.4%) were presumed to have IPA [18]. CAPA occurred after a median of 11.5 days (range 8–42 days) from COVID-19 symptom onset and after a median of 5 days (range 3–28) from ICU admission. A national, multi-center prospective cohort evaluation of a strategy to diagnose IFI in COVID-19 patients admitted to Welsh ICUs revealed an incidence rate of 25.9% (13.3% aspergillosis, 12.6% other yeast infections) [22]. The reported mortality rate was higher in patients with IFIs compared to those without fungal disease (51% vs. 31%, respectively, *p* = 0.039). Mortality reduction was associated with the use of antifungal treatment (38.5% vs. 90%, *p* = 0.008). Data from a prospective study, which included all the COVID-19 patients admitted to a tertiary hospital in Spain, showed that CAPA was diagnosed in 0.3% of the 2723 patients with COVID-19 hospitalized at that center, accounting for 3.3% of the 239 patients in the ICU. All patients were under mechanical ventilation and had received tocilizumab and corticosteroids [23].

### 2.2. Impact of IPA on Mortality in Patients with SARS-CoV-2 Infection

It is still difficult to determine how fungal co-infection impacts mortality. However, published data show the dramatic impact of IPA co-infection in influenza with mortality reaching 23% in some European centers [15,24]. White et al. evaluated the outcome of critically ill COVID-19 patients and reported a higher mortality rate in patients with IFIs compared to those without fungal disease (51% vs. 31%, respectively, *p* = 0.039) [22]. Mortality reduction was associated with the use of antifungal treatment (38.5% vs. 90%, *p* = 0.008).

It remains unclear whether COVID-19-associated pulmonary aspergillosis directly contributes to increased mortality rates or unequally affects the most severely ill patients who are burdened with comorbidities. The high heterogeneity in mortality among studies could be explained by the limited number of patients with CAPA and the differences in treatment strategies. In the study of Schauwvlieghe et al., the 3-month mortality rate of influenza was 51% when associated with IPA and 28% without IPA [14]. A retrospective study by Ku et al. described an increased risk of mortality among severe influenza patients with aspergillosis compared to severe influenza patients without Aspergillus co-infections [25]; in-ICU mortality of patients with Aspergillus co-infection was significantly higher than the mortality of patients with other coinfections (66.7% vs. 23.7%, *p* = 0.001) or control group without co-infections (15.4%, *p* < 0.001). However, data from a French cohort study did not demonstrate higher mortality rates in patients with IPA in comparison with COVID-19 cases without IPA [17]. A retrospective analysis using clinical data of 182 patients worldwide, who received a CAPA diagnosis between 1 March 2020 and 31 August 2020, comprising data from the FungiScope registry and academic literature, showed various cumulative incidence rates of CAPA in the ICUs ranging from 1.0% to 39.1% [26]. IPA was diagnosed in a median of ten days after coronavirus disease diagnosis (range 0–51 days). The study reported a high mortality rate of patients with CAPA admitted to the ICU (52.2%), while 33% of the deaths were attributed to CAPA.

Nonetheless, the true incidence of CAPA and its impact on mortality is difficult to define because of indiscriminate clinical signs, low culture sensitivity, specificity, and variability in clinical practice between centers [27]. An underestimation of the incidence of CAPA might be occurring due to the difficulties surrounding fungal infection diagnosis outside specific contexts [27]. Discrimination between colonization with *Aspergillus* spp. and IPA could be complicated. A recent study, discussing the differences and similarities between influenza-associated pulmonary aspergillosis (IAPA) and CAPA, concluded that compared to IAPA, the majority of CAPA cases could be categorized as putative rather than proven or probable IPA, due to the lack of histopathological evidence and positive galactomannan tests [28].

Undoubtedly, data from the literature indicate that patients with COVID-19 are at high risk for developing IPA. Diagnosis of CAPA can negatively impact the prognosis and subsequently increase the mortality rate among patients with COVID-19. In light of the epidemiological and mortality data, the recognition and appropriate treatment of patients with CAPA should be considered an essential component of an optimized approach to critically ill patients with SARS-CoV-2.

### 2.3. Risk Factors for IPA in Patients with SARS-CoV-2 Infection

SARS-CoV-2 patients undergo severe pulmonary damage caused by complex inflammatory processes, including the ensuing cytokine storm and the replication of the virus [29]. As with IAPA, the development of CAPA is rapid after ICU admission [13,14]. COVID-19 patients are predisposed to develop CAPA, due to the same risk factors as those that were identified for IAPA [28,30,31]. Lamoth et al. evaluated the similarities and differences between influenza-associated pulmonary aspergillosis (IAPA) and COVID-19-associated pulmonary aspergillosis (CAPA) [28] and concluded that the proportion of patients with immunosuppressive host factors predisposing to IPA appears to be higher among severe influenza patients compared to severe COVID-19 patients (approximately 25–30% vs. <10%, respectively); also, ARDS in COVID-19 was predominantly observed among a specific category of patients with no particular risk of IPA, especially those with hypertension, diabetes mellitus, and obesity. Among others, the major risk factors for CAPA are severe pulmonary damage due to the SARS-CoV-2, the widespread use of broad-spectrum antibiotics in ICUs, the use of corticosteroids in those with acute respiratory distress syndrome (ARDS), the presence of comorbidities, such as structural lung defects, older age, and male gender [17,18,19,22,32,33,34]. Additionally, this cohort is at particular risk, because of a combination of alterations in systemic immune function, the use of antimicrobial therapy, prolonged and invasive mechanical ventilation, and the presence of vast portals for infection via intravascular devices [35]. It is interesting that, even in immunocompetent patients with ARDS due to viral infections, the risk for IPA is increased [36]. Yet, non-immunocompromised patients developing SARS-CoV-2 suffered from at least one underlying comorbidity, such as diabetes, hypertension, chronic kidney disease, or chronic obstructive pulmonary disease (COPD), which predisposes them to IFIs [37,38]. Analysis of the frequent IFIs registered in the national hospital discharge database between 2001 and 2010 in France (total number of IFI cases 35,876), including candidemia (43.4%), *Pneumocystis jirovecii* pneumonia (26.1%), IA (23.9%), cryptococcosis (5.2%), and mucormycosis (1.5%), showed an increased risk of mortality from IFIs in patients with co-morbidities, ranging from 9.2% to 40% [7]. Moreover, this study demonstrated that candidemia and IA incidence was increased among patients with hematologic malignancies (more than 4% per year) and those with chronic renal failure (more than 10% per year). High incidence of IA was diagnosed in neutropenic patients, patients receiving chemotherapy, patients with prolonged corticosteroid therapy, hematopoietic stem cell recipients, solid organ recipients, or chronic respiratory disease patients [39]. It is noteworthy that the proportion of patients with immunosuppressive factors predisposing to IPA appears to be higher among severe influenza patients compared to severe COVID-19 patients [14,40,41].

The association of IA in patients with ARDS, after corticosteroid use, had previously been reported almost two decades earlier [42]. Corticosteroids are associated with improved outcomes in critically ill patients with SARS-CoV-2 infection [43,44,45,46]. Corticosteroid use is referred to as an important acquired immunological risk factor contributing to the risk of CAPA [47,48]. Wauters et al. reported that corticosteroid usage seven days prior to admission to ICU is an independent risk factor for IFI [13]; the week before ICU admission, the patients with IPA who received corticosteroids were significantly more than those who did not (78% vs. 23%, *p* = 0.002). In addition to that, corticosteroid dosages, before admission to the ICU, were significantly higher in IPA patients (*p* = 0.005). Multivariate analysis showed that corticosteroid usage, prior to the admission to the ICU, was independently associated with IPA (OR:14.4, CI:2.0–101.6, *p* = 0.007). Thus, it is evident that, especially in critically ill patients with COVID-19 who receive corticosteroids, the vigilance for IPA should be particularly high.

## 3. Diagnosis of IPA in Patients with SARS-CoV-2 Infection

### 3.1. Diagnostic Criteria

Early initiation of appropriate antifungal treatment remains a major predictor of outcomes in IFIs and is pivotal for successful treatment; however, many uncertainties exist regarding the identification and diagnosis of CAPA [28]. Early diagnosis of IFIs is still difficult, despite novel breakthroughs in diagnostic procedures, especially prior to the development of a typical radiological image. There is also an extreme difficulty in the differential diagnosis between the colonization by *Aspergillus* and IPA, especially in ICU patients. Thus, due to the absence of a ‘‘gold standard’’, the diagnosis of IPA remains a strenuous challenge, as it depends on clinical and microbiological data, along with histopathology when feasible [27].

Ideally, screening for CAPA includes the use of a combination of imaging methods (X-ray, CT scan) with *Aspergillus* antigen tests in bronchoalveolar lavage (BAL) and serum, including galactomannan (GM), lateral flow tests, or *Aspergillus* PCR tests [35,49,50,51]. However, the use of imaging and of other diagnostic methods must be balanced with the risks for other patients and healthcare workers during the process of obtaining samples, as well as for the patients themselves, during their transport and stay inside the CT scan room. Pronounced hypoxemia frequently prohibits the transferring of patients for diagnostic CT scans; BAL sampling also poses risks because of possible virus dispersion. Additional issues, which could complicate the diagnostic approach in COVID-19 disease, include a shortage of standard equipment for microbiological examinations and a lack of expert professionals to precisely identify the specific fungal infections [27,52].

**Figure 1:**
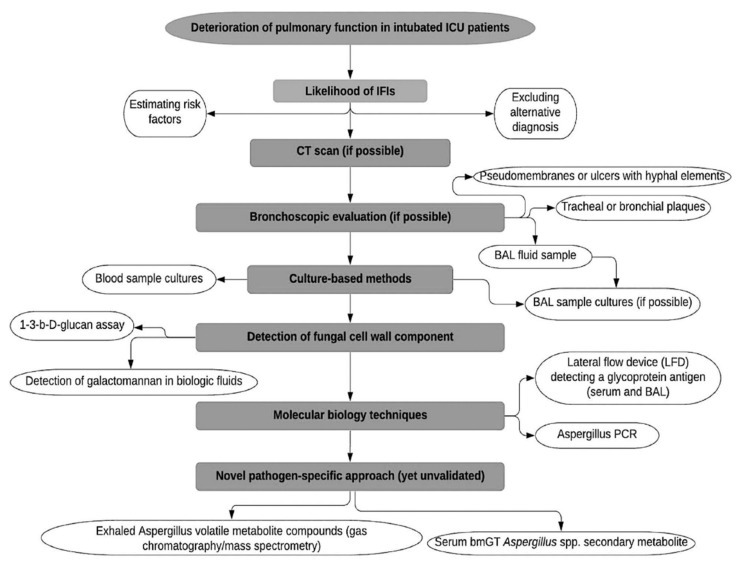
Diagnostic process of COVID-19-associated pulmonary aspergillosis. BAL: Bronchoalveolar lavage, ICU: Intensive Care Unit, IFIs: Invasive Fungal Infections, CT: Computed Tomography, LFD: Lateral Flow Device, PCR, Polymerase Chain Reaction.

Recently, a panel, including 29 international experts, reviewed current insights into the diagnosis and management of IAPA in ICU patients and proposed a case definition of IAPA, which would be appropriate to use in clinical studies, focusing on four main areas: (a) entry criteria, (b) host factors, (c) clinical features, and (d) mycological evidence of infection [27]. Firstly, in addition to a positive diagnostic test for influenza, patients would require having a clinical symptomatology compatible with influenza disease and respiratory distress syndrome during a timescale between one week before ICU admission and 72–96 h post-admission. Secondly, host factors referred to the EORTC/MSGERC definition and AspICU algorithm [47,53], were not considered as a key element of the diagnostic process and have not been included in the consensus definition for IAPA, despite the fact that most IAPA cases have at least one underlying condition, such as steroid use, diabetes mellitus or obesity. Thirdly, the authors pointed out, that the distinction between proven and probable IAPA is of utmost importance for clinical trials, while in clinical practice, clinicians should not distinguish between proven and probable disease. The authors reported tracheobronchitis as a separate entity, characterized by tracheal or bronchial ulcerations or nodules, the presence of hyphal elements suggestive of *Aspergillus* on pseudomembranes, or the presence of plaques, visualized during bronchoscopy. The proposed criteria for the proven disease include the fulfillment of the entry criterion, combined with histological evidence of invasive fungal elements, in biopsy or in brush specimens (airway plaques, pseudomembranes, or ulcers with hyphal elements) and mycological evidence for the presence of *Aspergillus* (*Aspergillus* growth on culture, or positive *Aspergillus* PCR in tissue). In patients with pulmonary infiltrates or endobronchial plaques, the diagnosis of probable IAPA should be confirmed by a positive GM test, obtained from a BAL sample, or positive culture of a sample from a tracheal aspirate. A serum GM index cutoff >0.5 and a BAL GM index cutoff ≥1.0 are recommended cutoff values that ensure high specificity and acceptable sensitivity, a fact that is also consistent with other recommendations [47,50]. A positive culture of an upper airway sample is considered indicative of the diagnosis of probable IAPA. However, the diagnosis should be confirmed with serum, BAL GM test, or positive BAL culture to mitigate the risk of overdiagnosis and over-treatment. In patients with tracheobronchitis, the presence of pulmonary infiltrates on chest X-ray, or other imaging methods, is not required to raise suspicion of probable disease. The basic steps of the diagnostic process of CAPA are presented in Figure 1.

Several fungal pathogens that cause invasive infections present similar morphology to *Aspergillus*, making its histopathological identification challenging. As a result, only the culture growth of the pathogen in question can definitively confirm the cause of the infection [54]. To make things worse, biopsy samples, which are necessary to achieve a diagnosis based on culture growth, are not easily obtainable in patients with SARS-CoV-2 and even when they are available, they do not always provide living microorganisms suitable for culture growth [36].

EORTC criteria for probable IFI include direct mycological tests, such as direct microscopy, culture or cytology, and indirect mycological tests, such as cell wall constituents, or antigen detection, as well as detection of β-D-glucan in serum, or of GM in serum, plasma, cerebrospinal fluid (CSF), or BAL [55].

Conventional microscopic examination and qualitative culture of respiratory tract samples have quite low sensitivity and specificity (around 50%) [39]. Additionally, respiratory tract cultures, even when obtained by BAL, may reflect airway colonization, and require a prolonged period of incubation, before yielding diagnostic data [56].

The spread of *Aspergillus* through vessels is a key characteristic of its pathogenesis, which allows the immunological tracking of the fungi via the detection of specific antigens in BAL or serum, namely, the galactomannan enzyme immunoassay (GM-EIA), and a ‘‘pan-fungal’’ assay, which detect *Aspergillus* GM and (1 → 3)-β-D-glucan, a preserved component of the fungal cell wall, respectively [54,57,58]. A prospective single-center study by Meersseman et al. investigated the role of GM in BAL fluid and serum, as a tool for early diagnosis of IA in the ICU; by using a cut-off index of 0.5, the sensitivity and specificity of GM detection in BAL fluid was 88% and 87%, respectively. In comparison, the sensitivity of serum GM was only 42% [59]. In 11 out of 26 proven IA cases, BAL culture and serum GM remained negative, whereas GM in BAL was positive. The authors concluded that GM detection in BAL fluid seems to be useful in establishing the diagnosis of IA in the ICU settings. In the retrospective multicenter cohort study by Schauwvlieghe et al. [14] that included adult patients with severe influenza admitted to seven ICUs across Belgium and the Netherlands, serum GM testing performed better with 20/31 positive cases (65%), nevertheless, BAL GM remained superior with 67/76 positive cases (88%). Rutsaert et al. [34], in a small study on CAPA, acquired bronchial aspirates or bronchoscopy-guided biopsies of suspicious lesions while performing bronchoscopic procedures due to various causes, such as respiratory deterioration or atelectasis. Subsequently, GM assays on BAL and serum were routinely assessed. IPA was diagnosed via histopathology in four patients all of whom presented positive GM in BAL but negative in serum (<0.5), concluding that the BAL GM test is probably superior to that of serum in the diagnosis of CAPA. Koehler and colleagues described IA in five out of nineteen patients admitted to their ICU (26%); three patients were identified as positive for *Aspergillus* spp. with PCR and GM from a BAL sample, one patient grew *Aspergillus* spp. on a tracheal aspirate, but was negative for serum GM and the final patient had positive serum GM with no growth on a tracheal aspirate [19]. Alanio and colleagues described nine out of twenty-seven SARS-CoV-2 patients (33%), admitted to their ICU, as having IA [17]. However, only one patient, with concurrent candidemia (*C. glabrata*), received antifungal treatment with voriconazole. Supportive diagnostic criteria, including serum GM and BAL GM, were negative in all patients and no deaths were attributed to IFI.

It has been reported that serum GM detection for the diagnosis of IA in COVID-19 patients is less sensitive than in influenza patients and GM testing is not sufficiently validated for upper respiratory tract samples [60]. A positive serum GM result (≥0.5) would be highly suspicious for CAPA, although a negative one should not be used to exclude the diagnosis [35,59].

Next-generation monoclonal antibody (MAb)-based assays were recently developed due to the problematic accuracy of the indirect tests. By using hybridoma technology, these assays detect Mab specific for *Aspergillus*. They have been used in the development of an immuno-chromatographic lateral flow device (LFD) for the diagnosis of IPA in the point-of-care (POC) [61]. The LFD test specific for *Aspergillus* is based on the JF5 Ab and detects an antigen that is a glycoprotein secreted extracellularly during active growth of *Aspergillus* spp. Since MAb binds to an extracellular substance which is secreted solely during fungus multiplication, this test provides the advantage of detecting only active strains. The LFD presented increased sensitivity and specificity compared to the β-D-glucan and GM assays, proving its usefulness in the diagnosis of IPA in various studies [54,61]. In addition to that, a similar monoclonal Ab476-based LFD for urine antigen detection has also been manufactured, although it requires additional validation [62,63].

Recent recommendations of the American Thoracic Society Assembly on pulmonary infections and tuberculosis stated that in immunocompromised adult patients who are suspected of having IPA the use of blood or serum *Aspergillus* PCR testing is recommended (strong recommendation, high-quality evidence) [50]. In patients with severe immunocompromising conditions, the recommendations suggest the inclusion of *Aspergillus* PCR in BAL testing as part of the evaluation (strong recommendation, high-quality evidence).

There is no clear evidence on how the empirical use of antifungal therapy in critically ill patients impacts PCR test performance since PCR can detect very low copy numbers. While the use of antifungal drugs seems to reduce the sensitivity of GM testing for IPA, the ability of PCR to detect low copy numbers makes it, possibly, an attractive option for assessing patients who receive active antifungal therapy. However, the high sensitivity of BAL-PCR makes it difficult to discriminate between IPA and simple *Aspergillus* colonization [50]. Furthermore, during bronchoscopy, an aerosol is developed, making it a hazardous procedure for viral contamination in COVID-19 units. As a result, it has been suggested that it should only be used when a definite diagnosis is required to change clinical management and samples obtained from the upper respiratory tract are negative [64]. In such cases, the ratio between the risk of viral transmission and the benefit of achieving the optimal diagnosis should be balanced in order to attain the best possible patient care.

Novel diagnostic biochemical markers, based on the detection of metabolites of *Aspergillus* spp. had recently been introduced. Filamentous fungi, including *Aspergillus species,* can produce an array of secondary metabolites, many of which are volatile [65]. These volatile organic compounds (VOCs) could identify evidence of *Aspergillus* metabolism in the breath of patients with IA [36,66,67]. Gliotoxin (GT), a secondary metabolite of *Aspergillus fumigatus,* and bis(methylthio)gliotoxin (bmGT), a degradation product of gliotoxin, have been proposed as potential biomarkers for IPA diagnosis [36,66]. However, recently published data showed a very poor performance of these biomarkers for diagnosing IPA [68], a fact that is not supportive of the use of serum or BAL GT/bmGT in routine practice.

Varying diagnostic performance, availability, and time-to-results turnaround time are important limitations of currently approved biomarkers and molecular assays for the diagnosis of IA. Specific characteristics of different diagnostic tests for CAPA are presented in Table 1.

### 3.2. The Role of Diagnostic Radiology

Differentiating between Aspergillus colonization and IPA is notoriously difficult, especially in the ICU setting. In the absence of host factors and diagnostic criteria, as defined by the EORTC, invasive or high-risk diagnostics (biopsy or CT scan) are required, to support the diagnosis of IPA [55]. However, the radiologic findings associated with IA are non-specific and often represent other IFIs such as mucormycosis or different nonfungal diseases, such as bacterial pneumonia, cryptogenic organizing pneumonitis (COP)*,* or even hemorrhage [88].

Unarguably, the diagnostic process for CAPA should include *Aspergillus* antigen tests from serum and BAL, including enzyme-linked immunosorbent assay (ELISA), LFD, GM, or Aspergillus PCR, along with chest CT imaging, since nodules with halo sign or other characteristic features of IA on chest CT were seen in 17.6% of COVID-19 patients with severe disease, but was not confirmed to be IPA. This is in accordance with the absence of classic chest CT characteristics of IAPA. Consequently, the lack of typical features, such as cavities, should not exclude CAPA. On the other hand, the presence of such features should support the diagnosis and reduce the number of further laboratory examinations [35].

Due to severe life-threatening hypoxia and challenges in mechanical ventilation, CT scanning is not considered possible for many patients with SARS-CoV-2. When performed, the differentiation between COVID-19 and *Aspergillus*-associated lesions could additionally be proved extremely complex [34]. Moreover, patient transfer to CT in these cases is often resource intensive. Clinical justification of CT procedures should be made on a local level, and CT should be reserved for cases where healthcare team discussion highlights a clear clinical indication.

### 3.3. Diagnostic Challenges, Summary

- In light of the current difficulties and uncertainties relating to the diagnosis and the risks associated with IA in COVID-19 patients, clinicians should maintain a high level of suspicion for this infection, especially in ICU patients;
- The persistence of a respiratory co-infection in SARS-CoV-2 patients despite the administration of broad-spectrum antibiotics should lead to the pursuit of the confirmation or exclusion of IPA with culture- and non-culture-based methods, especially in those patients who present risk factors for IPA;
- Bronchoscopy has a limited role in these patients and should only be considered when diagnosis confirmation would significantly change clinical management;
- Conventional microscopic examination and qualitative culture of respiratory tract samples have quite low sensitivity and specificity;
- Confirmation test with blood biomarkers (serum GM or beta-D-glucan), blood PCR, or BAL GM or PCR, if possible, could be performed in cases of high clinical suspicion;
- The use of CT scans for diagnostic purposes is controversial due to practical concerns and the complex character of lesions presented in SARS-CoV-2 patients;
- Implementation of immuno-chromatographic LFD for the POC diagnosis of IPA could be helpful.

## 4. Challenges in the Treatment of IPA in Patients with SARS-CoV-2 Infection

Despite the available treatment options, the mortality rate of IA in non-neutropenic patients remains extremely high (up to 90%) [66,89,90]. Experimental and clinical data indicate that delayed initiation of antifungal therapy could be detrimental in IA [91,92].

Patients admitted to the ICU with a high risk of IA (i.e., patients with malignancies, COPD, patients receiving prolonged treatment with steroids or other immunosuppressive drugs, those receiving steroids and immunosuppressive therapy as part of COVID-19 therapy, patients with hepatic or renal failure, and ICU-related immunoparalysis) should receive adequate antifungal therapy upon suspicion of IA, even in the absence of definitive diagnosis of infection. Whenever possible, a CT scan of the lower respiratory tract, fungal cultures, and a combination of serological biomarkers (GM or 1,3-b-D-glucan assay) or *Aspergillus* PCR test should be performed and treatment should be reevaluated and stopped if the diagnosis of IA is not confirmed [66,93].

While it is currently unknown whether antifungal treatment of CAPA translates into an outcome benefit, the diagnosis should trigger early antifungal treatment. To date, antifungal agents recommended as first-line treatment options for IPA include voriconazole and isavuconazole or amphotericin B and its lipid formulations [49,51,66]. Data from the literature describing the treatment of COVID-19 patients co-infected with *Aspergillus* species show that the most commonly used drugs are voriconazole, liposomal amphotericin B, caspofungin, the combination of isavuconazole and voriconazole with anidulafungin and combination of voriconazole with isavuconazole [93]. The main factors that should be considered upon the selection of the treatment option include the severity of the infection, renal or hepatic injury, possible drug interactions, the requirement for therapeutic drug monitoring, and the cost of therapy [66,93,94].

Voriconazole is currently regarded as a drug of choice for the treatment of proven or probable IPA with a high confirmed treatment response rate [95,96]. Recently published guidelines by the European Society for Clinical Microbiology and Infectious Diseases (ESCMID), the European Confederation of Medical Mycology (ECMM), and the European Respiratory Society (ERS), as well as the Clinical Practice Guidelines of the Infectious Diseases Society of America (IDSA) [49,51] considered triazoles as drugs of choice for the primary treatment and prevention of IA in most patients because of reduced mortality related to voriconazole treatment (strong recommendation, high-quality evidence, IDSA recommendations). However, the narrow therapeutic window of voriconazole and the requirement for therapeutic drug monitoring to guarantee efficacy and prevent neurotoxicity, hepatotoxicity, and drug–drug interactions may constitute the main limitations for its use in the ICU setting [49,90]. Being metabolized via oxidation by the hepatic cytochrome P450 (CYP) isoenzymes, CYP2C19, CYP2C9, and CYP3A4, voriconazole is among the drugs most frequently associated with a wide range of drug–drug interactions. Interactions with experimental COVID-19 treatment drugs, including hydroxychloroquine, atazanavir, lopinavir/ritonavir, and remdesivir have recently been described [97]. Additionally, the inhibition or induction of CYP450 enzymes may alter the pharmacokinetic profile of the drugs involved and can therefore affect the interacting agents [98]. Voriconazole demonstrates wide interpatient variability in serum concentrations. Polymorphisms in CYP2C19 contribute to the variability of voriconazole pharmacokinetics, thus, therapeutic drug monitoring has become the standard of care to ensure efficacy and avoid adverse effects [99,100]. The majority of studies investigating the impact of voriconazole drug monitoring on efficacy and safety have found this approach to be beneficial, leading to an increased probability of a successful outcome and preventing drug-related toxicity and the emergence of drug resistance [49,99,101].

Liposomal amphotericin B and Isavuconazole are the main alternative treatment options for IPA in ICU [49,51]. Isavuconazole demonstrates fewer adverse effects and a more favorable pharmacokinetic profile compared to voriconazole [49,102,103]. Liposomal amphotericin B is an effective alternative treatment option and may replace voriconazole as first-line treatment in areas or institutions with a high prevalence of azole resistance [49]. However, the co-existence of severe renal or hepatic failure in ICU patients with SARS-CoV-2 often prevents initiation or leads to discontinuation of this antifungal agent [93,104,105,106].

Echinocandins present limited activity against *Aspergillus* spp.; therefore, they do not constitute a primary therapeutic choice for IA. Yet, they demonstrate static activity against *Aspergillus* hyphae, limited interactions with other drugs and they are generally well tolerated [107]. Echinocandins are considered efficacious against *Aspergillus* spp., both in vivo and in vitro. Nevertheless, caspofungin is the only one which is approved for IA treatment in those who are intolerant to first-line antifungal therapy. A combination of antifungal therapy can be considered as a choice in refractory disease (e.g., echinocandin plus liposomal amphotericin B, or voriconazole) [51,66,107].

New antifungal drugs currently under development (fosmanogepix and olorofim) [108] may have equivalent efficacy without exhibiting the same spectrum of drug interactions and toxicity in comparison to currently available drugs. Rezafungin, a novel echinocandin with exceptional stability and solubility and a uniquely long half-life could be another addition to the antifungal drug armamentarium for prophylaxis and treatment of invasive aspergillosis [109].

The adequate duration of antifungal therapy for IPA in patients with COVID-19 disease is still under discussion. The IDSA guidelines recommend the treatment duration for IPA to be continued for a minimum of 6–12 weeks [51], depending on the clinical condition of the patients, as well as the time course and the degree of clinical resolution of the disease. Careful clinical evaluation, estimation of specific biomarkers, and imaging are crucial for determining the therapeutic response and the length of treatment.

The appropriate use of antimicrobial agents improves clinical outcomes and reduces antimicrobial resistance. Nevertheless, the data on inappropriate prescription of antifungal treatment and negative outcomes are inconsistent. Aldrees et al. performed a retrospective chart review for patients who received antifungal treatment. The appropriateness of the dosage, initiation time, agent selection, and duration of therapy was evaluated based on international recommendations [110]. Overall, 270 (76.1%) patients received empirical treatment, 56.3% of which had received antifungal treatment for more than five days despite the absence of proven fungal infection. Only 39% of patients who were subjected to antifungal therapy met all study criteria for an appropriate prescription. A recently published study by Estella et al. investigated the impact of early anticipatory antifungal treatment on the incidence of CAPA and outcomes of critically ill patients with pneumonia [111]. There was a comparison between the two analysis periods based on whether antifungal therapy had been initiated early or late. The results of the study demonstrated that early initiation of antifungal therapy was associated with a decrease in the incidence and mortality of pulmonary aspergillosis. Conflicting data in the literature regarding the appropriate prescription of antifungals mandate the use of antimicrobial stewardship programs which can improve the prompt utilization of antifungal therapies.

Another growing concern about the management of CAPA is the high variability of plasma concentration in COVID-19 patients, especially in those treated with ECMO [112,113]. Both subtherapeutic and toxic levels have been detected in critically ill COVID-19 patients, resulting in a higher probability of neuro- and hepatotoxicity or therapeutic failure [112]. Additionally, a delay was observed in reaching voriconazole therapeutic levels (2–6 mg/L) in CAPA patients, with Reizine et al. demonstrating that the therapeutic range was achieved at day 7, with 83.3% of CAPA patients having subtherapeutic levels [114]. Dexamethasone, the primary treatment for severe COVID-19, may be involved by activating various CYP450 enzymes and reducing plasma voriconazole concentrations. [115].

Immunosuppressive and immunomodulatory treatment strategies using drugs that reduce the level of inappropriate systemic inflammation (anakinra (interleukin-1 receptor antagonist) or Janus kinase (JAK) inhibitors) seem to be an attractive approach [116]. However, over-suppression of the immune system caused by this specific treatment might favor the rise of potential opportunistic fungal infections. Further studies are required to confirm and validate the safety and efficacy of immunotherapy in patients with COVID-19.

### Treatment Challenges, Summary

- Key objective is to improve survival, by avoiding misdiagnosis and by initiating early, targeted, and specific antifungal treatment. Any patient at risk should be considered by the responsible clinician as having IA and should receive antifungal therapy;
- There are possible drug–drug interactions between antifungal agents and agents used for specific treatment of coronavirus infection (tocilizumab-IL-6 receptor blocker-anakinra);
- The antifungal drug arsenal is very limited with high toxicity and severe side effects;
- Prolonged exposure to novel echinocandins (e.g., anidulafungin, micafungin), or triazoles (e.g., voriconazole, isavuconazole, and posaconazole) may result in the development of new resistance patterns leading to treatment failures;
- Lack of necessary equipment for microbiological examination, failure of early detection of fungal growth in infected tissue, incorrect technique of specimen sampling and clinicians’ failure to identify the precise fungi lead to high mortality rates;
- The optimal duration of antifungal therapy for CAPA is still under debate;
- Over-suppression of the immune system caused by the disease or the use of specific trial treatment (anakinra-recombinant IL-1Ra- or Janus kinase (JAK) inhibitors), might favor the rise of potential opportunistic fungal infections.

## 5. Conclusions

The global burden of fungal diseases is increasing, given the expanding number of SARS-CoV-2 patients at risk for these infections. Recognizing and appropriately treating COVID-19 patients with IPA is considered an essential component of an optimized approach to patients with SARS-CoV-2. The growing wave of patients with COVID-19, the complicated medical situations of the disease, and the high pressure on the healthcare systems may contribute to the difficulties in the identification of IPA. Given the expanding population of COVID-19 patients, who are at higher risk for fungal disease, early diagnosis could provide the best chance for targeted treatment. Prospective studies are urgently required, to provide precise insight into the risk factors and potential outcome of aspergillosis in COVID-19 and to support evidence-based recommendations on diagnosis and treatment.

## Figures and Tables

**Table 1:** Diagnostic tests for CAPA, features and pitfalls [36,49,50,51,55,56,63,66,68,69,70,71,72,73,74,75,76,77,78,79,80,81,82,83,84,85,86,87].

Tests	Features	Diagnostic Value	Turnaround	Pitfalls
Conventional microscopic examination [36,49,50,51,69,70]	Availability. Simplicity. Low cost.	Suboptimal, low to moderate sensitivity and predictive value.	Rapid.	Challenging to differentiate between infection and colonization. May reflect airway colonization.
Respiratory sample cultures [49,50,51,56,70]	Simplicity. Low cost. Identification of species. Antifungal susceptibility testing.	Suboptimal, low to moderate sensitivity and predictive value.	Prolonged.	Challenging to differentiate between infection and colonization.
Galactomannan (GM) in biologic fluids [36,49,51,55,69,71,72,73,74,75]		Serum: Low or moderate sensitivity depending on the index cut-off used. Moderate specificity. Better performance in neutropenic than in non-neutropenic patients. BAL: Moderate or high sensitivity and high specificity of 81–96.6% depending on the optical density index cut-off used, sensitivity exceeds 70% in most studies. Raising the cutoff improves test specificity without compromising sensitivity. High NPV, moderate or high PPV.	Variable.	Variable performance. BAL: Optimal threshold has not been determined; sensitivity may be reduced in the presence of antifungals.
Serum 1-3-b-D-glucan (BDG) assay [36,49,51,66,76]		Low or moderate sensitivity (49.6–80%), good specificity (82–98.9%), acceptable PPV (83.5%), high NPV (89–94.6%) (useful to exclude diagnosis rather than confirm it).	Variable.	False-positive results (b-lactam antibiotics, human blood products, immunoglobulin, albumin plasma, cellulose hemodialysis membranes, bacterial bloodstream infections, e.g., Pseudomonas aeruginosa)
PCR-based methods [36,49,51,70,77,78,79,80,81,82]	High cost. Not affected by the immune status of the patients. Evaluation of phenotypes of strains.	Heterogeneity of results. High NPV. Two positive consecutive results have high specificity and high positive likelihood ratio, single negative PCR result has high NPV. High sensitivity in combination with other fungal biomarkers in serum (either GM or BDG) or in BAL and along with GM and/or LFD test.	Rapid.	Requires further clinical standardization. Potential for contamination due to the environmental ubiquity of fungal nucleic acids.
*Aspergillus*-specific immuno-chromatographic lateral flow device (LFD) test [36,49,51,63,66,74,83]		Acceptable sensitivity, specificity, moderate PPV, high NPV (especially in combination with BAL GM) [66,84].	Rapid.	Requires further clinical evaluation. Sensitivity of the BAL LFD assay may be reduced in the presence of antifungal treatment.
Novel assays: volatile organic compounds (VOC) assays, Gliotoxin (GT), bis(methylthio)gliotoxin (bmGT) assays [67,68,85,86,87]		High sensitivity and specificity. bmGT presents higher sensitivity and PPV than GM and similar specificity and NPV. Importantly, the combination of GM and bmGT increased the PPV (100%) and NPV (97.5%) of the individual biomarkers.	Rapid.	Requires further clinical evaluation.

CAPA: COVID-19-Associated Pulmonary Aspergillosis, GM: Galactomannan, BAL: Bronchoalveolar lavage, NPV: Negative Predictive Value, PPV: Positive Predictive Value, BDG: b-D-glucan, PCR: Polymerase Chain Reaction, LFD: Lateral Flow Device, VOC: Volatile Organic Compounds, GT: Gliotoxin, bmGT: bis(methylthio)gliotoxin.

## Data Availability

No new data were created or analyzed in this study. Data sharing is not applicable to this article.

## Appendix A

**Table A1:** Abbreviations expanded.

Abbreviation	Expansion
ARDS AspICU	Acute Respiratory Distress Syndrome Clinical Algorithm to Diagnose Invasive Pulmonary Aspergillosis in Critically Ill Patients (by Blot et.al., ref. [53])
BAL	Bronchoalveolar Lavage
BDG	b-D-glucan
bmGT	bis(methylthio)gliotoxin
CAPA	COVID-19-Associated Pulmonary Aspergillosis
CDC	Centers for Disease Control and Prevention
COP	Cryptogenic organizing pneumonitis
COPD	Chronic Obstructive Pulmonary Disease
COVID-19	Coronavirus Disease 2019
CSF	Cerebrospinal Fluid
CT	Computed Tomography
CYP	Cytochrome P
ECMM	European Confederation of Medical Mycology
ELISA	Enzyme-Linked Immunosorbent Assay
EORTC	European Organization for Research and Treatment of Cancer
ERS	European Respiratory Society
ESCMID	European Society for Clinical Microbiology and Infectious Diseases
GAFFI	Global Action Fund for Fungal Infections
GM	Galactomannan
GM-EIA	Galactomannan Enzyme Immunoassay
GT	Gliotoxin
HIV	Human Immunodeficiency Virus
IA	Invasive Aspergillosis
IAPA	Influenza-Associated Pulmonary Aspergillosis
IDSA	Infectious Diseases Society of America
IFIs	Invasive Fungal Infections
IPA	Invasive Pulmonary Aspergillosis
JAK	Janus Kinase
LFD	Lateral Flow Device
Mab	Monoclonal Antibody
MSGERC	Mycoses Study Group Education and Research Consortium
OR	Odds Ratio
PCR	Polymerase Chain Reaction
POC	Point-Of-Care
SARS-CoV-2	Severe Acute Respiratory Syndrome Coronavirus 2
